# Central nervous system nocardiosis in a 54-year-old man with lateral ventricular choroid plexitis and diffused meningitis: A case report

**DOI:** 10.1097/MD.0000000000039198

**Published:** 2024-08-02

**Authors:** Qiujian Yu, Shujuan Dai, Ailan Pang

**Affiliations:** a Department of Neurology, First Affiliated Hospital of Kunming Medical University, Kunming, China.

**Keywords:** case reports, central nervous system infections, choroid disease, deep sequencing, meningitis, Nocardia infections

## Abstract

**Introduction:**

Nocardiosis is an unusual infection caused by aerobic gram-positive bacteria in the genus *Nocardia*. Infections resulting from *Nocardia* species are frequent in immunosuppressive patients. Weakened immune systems caused by human immunodeficiency virus infection, diabetes, cancer, and other conditions such as chronic lung disease, renal failure, etc, are the main risk factors for nocardiosis. Central nervous system (CNS) nocardiosis has been reported to represent ~2% of all and to be present in 15% to 50% of patients with systemic infection. The patient in our case had an isolated CNS nocardiosis caused by *Nocardia terpenica* infection, a rare reclassified *Nocardia* pathogen of CNS nocardiosis.

**Case:**

We here present a 54-year-old Chinese male with a fever and headache for 15 days who showed positive meningeal irritation signs. Magnetic resonance imaging showed the right trigone of the lateral ventricular choroid plexitis and diffused leptomeningeal meningitis involving the bilateral cerebral hemisphere, cerebellar hemisphere, and brain stem. The patient was quickly diagnosed with CNS *Nocardia* infection by next-generation sequencing within 48 hours after admission. Meanwhile, the diagnosis was validated by *Nocardia*-positive staining in cerebral spinal fluid culturing. The patient was given trimethoprim-sulfamethoxazole, and his symptoms recovered after 3 days.

**Conclusions:**

In this case, the clinical, radiological, and microbiological findings highlight the importance of suspecting *Nocardia* as the potential pathogen in patients with central nervous system inflammation of doubted immune incompetence. In addition, next-generation sequencing as an effective test is also highly recommended for suspicious CNS infection patients to perform a rapid diagnosis and treatment.

## 1. Introduction

It is well-known that neuroinfectious diseases are one of the most devastating illnesses. Depending on the agent, age, and immune status of the infected host, different symptoms are presented, and in some cases, leading to up to 100% mortality.^[1]^ Speaking of the various types of infectious pathogens, those within the expansive categories of bacteria, viruses, fungi, and parasites lead to central nervous system (CNS) inflammation of the meningeal or parenchymal compartments. *Nocardia* species can be found worldwide, and human nocardiosis infections have been reported on all continents except Antarctica.^[2]^ It has been reported that approximately 20% to 58% of CNS infections are caused by nocardial infection,^[3]^ and most of the cases are reported in immunocompromised individuals.^[4]^ Risk factors that cause CNS nocardiosis are weakened immune systems and other conditions. More precisely, patients with human immunodeficiency virus infection,^[5]^ organ transplantation,^[6]^ primary immune deficiency,^[7]^ and cancer^[8]^ are prone to opportunistic infections. Other conditions, such as chronic lung disease,^[9]^ diabetes,^[10]^ tuberculosis,^[11]^ etc, are the most seen underlying diseases that lead to CNS nocardiosis infections. For the clinical features that are related to CNS nocardiosis, most patients suffer from brain obsess, and fewer are manifested as meningitis.^[12]^ Here, we present a patient with lateral ventricular choroid plexitis and diffused leptomeningeal meningitis under the suspicion of immune deficiency. He was diagnosed with CNS nocardiosis within 48 hours after admission by next-generation sequencing. After receiving sulfamethoxazole (SMX), his symptoms were recovered within 3 days.

## 2. Case presentation

A 54-year-old right-handed Chinese male, without engaging in any special type of work, presented to the Department of Neurology of the First Affiliated Hospital of Kunming Medical University for evaluation of recurrent fever and headache over a period of 15 days. Fifteen days ago, he initially noticed a running nose accompanied by pain and discomfort in his limbs. Then, he recovered after receiving unknown treatment in the local hospital. The other day, he found himself with a sudden headache and fever (up to 38.8°C of body temperature). Accompanied by nausea. There were no seizures, consciousness disorders, cognitive decline, visual changes, or ataxia in the course of the disease.

Additionally, he had poor spirit, diet, and sleep but with normal stool and urine and no change in weight. The patient is a known diabetic and hypertensive for the last 6 months. His blood pressure and blood sugar were controlled normally by taking antihypertensive and antidiabetic drugs regularly. He had rheumatoid arthritis (RA) for more than a year and was granted unknown herbal medicine for the treatment. There was no other previous history and no known family history. He had neck rigidity and a positive Kerning sign for his physical examination.

**Figure 1.**
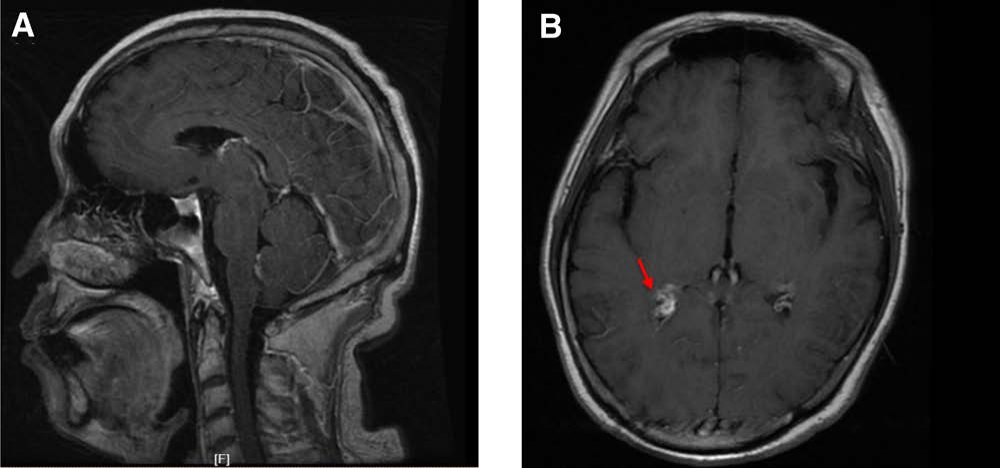
Brain magnetic resonance image (T1-weighted sequence with gadolinium). The sagittal sequence shows the leptomeningeal of the bilateral cerebral hemisphere, cerebellar hemisphere, and brain stem meninges were slightly thickened and enhanced (A). From the axial sequence, the right trigone of the lateral ventricle showed a gadolinium-enhanced lesion (B).

In our case, laboratory evaluation showed a white blood cell count of 15.53 × 109/L, 85.9% neutrophils, 7.3% lymphocytes, and 6.1% monocytes. The patient had mild anemia with a red blood cell count of 3.94 × 1012/L and hemoglobin of 92 g/L. The results of rheumatoid-related antibodies, including rheumatoid factor IgA/IgG/IgM and anticyclic citrullinated peptide antibody, were considerably increased than normal standards. The patient also showed elevated levels of C-reactive protein (34.3 mg/L) and ESR (48 mm/h). A lumbar puncture was performed and showed white blood cells in 1278 × 106/L (59% neutrophils and 29% lymphocytes), no red blood cells, glucose 1.47 mmol/L (blood sugar 5.8 mmol/L), and total protein 1.19 g/L. The opening pressure was 160 mm H_2_O. Since this patient was admitted to the hospital during the pandemic, we have carefully detected the patient’s severe-acute-respiratory-syndrome-related coronavirus antigen, and the result was negative. An enhanced magnetic resonance imaging of the brain was performed, revealing that the bilateral cerebral hemisphere, cerebellar hemisphere, and brain stem meninges were slightly thickened and enhanced. Meanwhile, an enhanced abnormal signal was presented in the right trigone of the lateral ventricle, which we believed is the choroid plexitis as shown with the red arrow (Fig. 1).

**Figure 2.**
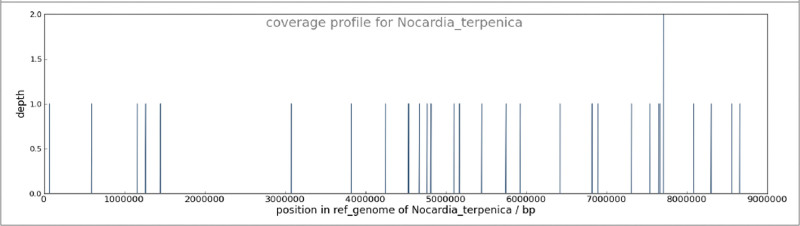
Coverage profile for *Nocardia terpenica*. Diagnosis of *Nocardia* infection by means of unbiased NGS shows the position in the reference genome of *N terpenica*. NGS = next-generation sequencing.

**Figure 3.**
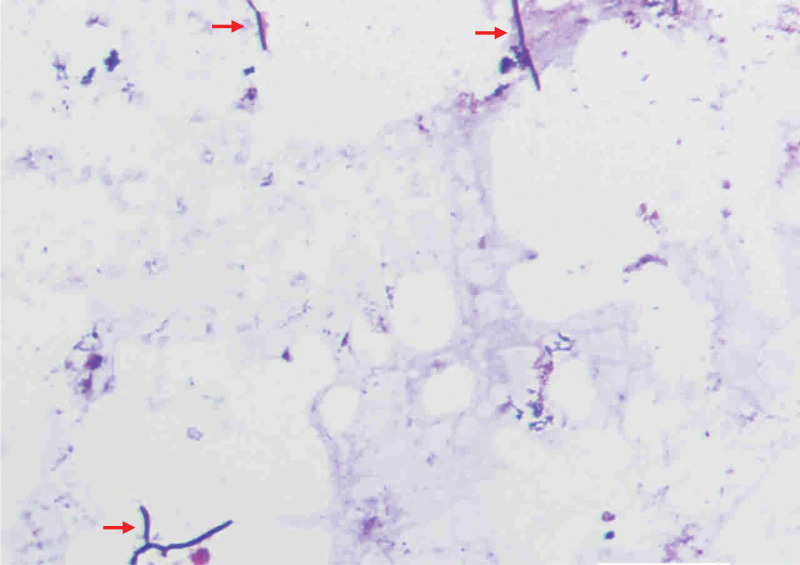
CSF bacterial culturing findings. Microscopy revealed the presence of nocardiosis after CSF culturing, as shown in red arrows. CSF = cerebral spinal fluids.

After being approved by the patient and his relatives, an analysis of the patient’s clinical samples (cerebral spinal fluid [CSF]) to identify potential pathogens was sent to Boao Genetic Testing Technology Center (Beijing, China). As previously described, the amplified DNA libraries for next-generation sequencing were constructed from extracted nucleic acid derived from clinical samples.^[13]^ Meanwhile, we also performed CSF culturing to validate the consequences of the next-generation sequencing. Within 48 hours after receiving the samples, next-generation sequencing analysis of more than 8 million sequences with the use of a bioinformatics pipeline for the detection of all known pathogens detected sequence reads corresponding to *Nocardia terpenica* in the patient’s CSF (Fig. 2). We then quickly responded to the result and administrated the patient orally with trimethoprim-SMX (TMP-SMX), for a dose of 40 milligrams (mg) per kilogram of body weight of SMX and 8 mg per kilogram of body weight of trimethoprim, every 12 hours. On the other hand, the identification of *Nocardia* was confirmed with CSF culturing in an independent laboratory after 1 week of admission (Fig. 3). After 3 days of taking medicine, the patient’s body temperature turned back to normal (36.4°C–36.8°C), and his headache significantly relieved, accompanied by the disappearance of the neck resistance. He was then discharged after 7 days and was asked to take the medicine for at least 6 months. Unfortunately, he didn’t follow up afterward due to the economic problem.

## 3. Discussion

*Nocardia* spp. is a specific pathogen associated with a variety of clinical infectious diseases. It is found in the infections of skin, soft tissue, respiratory tract, and even the CNS. Infections caused by *Nocardia* species often occur during periods of immunosuppression mostly, such as those that occur with underlying malignancy, chronic granulomatous disease, systemic lupus erythematosus, human immunodeficiency virus infection, organ transplantation, surgery, or long-term corticosteroid use.^[14]^ The most common neurological manifestations in the course of nocardiosis are altered mental status, focal neurologic abnormalities, visual changes, seizures, and ataxia. Nocardial meningitis is an infrequent manifestation of CNS nocardiosis and can occur with or without an associated brain abscess.^[15]^ In our case, the patient initially had only a headache and fever, which could be easily neglected. Therefore, the neurological examination is essential since the patient showed remarkably meningeal irritation. Diffused meningitis from the patient’s neuroimaging can help to explain the patient’s neck rigidity and positive Kerning sign.

Interestingly, in this case, we found an asymmetrical enhancement of patient’s choroid plexus, which we believed was choroid plexitis. Choroid plexitis is a general term defined as an infectious process affecting the choroid plexus. It is commonly accompanied by encephalitis, meningitis, or ventriculitis. The choroid plexus can also be a location of noninflammatory conditions, such as sarcoidosis, xanthogranulomas, and rheumatoid nodules.^[16]^ Here, our patient had a 1-year history of RA. It is important to make a differential diagnosis between infection-caused choroid plexitis and rheumatoid nodules. The patient didn’t have the main clinical symptoms of RA such as joint pain and swelling, joint stiffness, and decreased movement.^[17]^ In addition, the patient was taking unknown herbal medicine regularly to control RA. Furthermore, his magnetic resonance imaging showed widespread meningitis with the involvement of the bilateral cerebral hemisphere, cerebellar hemisphere, and brain stem. Based on these clinical findings, we’re more inclined to think he got choroid plexitis caused by infection rather than rheumatoid nodules. It is unnecessary to perform neuro-biopsy, although it is the golden standard for the diagnosis.^[18]^

For the mechanisms of CNS infection, it has been reported that pathogens like *Nocardia* spp. can attack the brain parenchyma or meningeal, affect multiple neural cell types, leading to inflammation dysfunction of the blood-brain barrier, cause local damage, and finally lead to cell death. Moreover, immunocompromised patients are prone to CNS infection localized in focal or diffused lesions.^[19]^ In our case, it is fair to suspect that the unknown herbal medicine that the patient took for treating RA contains corticosteroids. A long-term use of corticosteroids might lead to immunosuppression, and CNS infections tend to be more rapidly progressive in patients with immunodeficiency than in persons with normal host defenses.^[20]^

The classified pathogen in this case is *N terpenica* showed by next-generation sequencing*. N terpenica* was reclassified from *Nocardia brasiliensis* in 2007. It is one of the rare species of *Nocardia*, consisting of 2 strains.^[21]^ Up to date, only a few cases of CNS nocardiosis have been found caused by *N terpenica* with the only presentation of cerebral abscesses.^[22]^ However, in this case, our patient had diffused meningitis and choroid plexitis without brain abscesses. In addition, this patient had no other system involvement, which is considered to be an isolated CNS infection.

CNS nocardiosis causes an average of fatality ~30%, especially in patients with immunodeficiency. The recommended treatments for CNS nocardiosis are antimicrobials, surgery, or both.^[23]^ TMP-SMX is the most used antimicrobials as the fundamental treatment in CNS *Nocardia* infection. Besides, other effective antimicrobials against *Nocardia* spp. such as meropenem, linezolid, moxifloxacin, imipenem, etc, are also used in patients from the literature.^[24]^ Fortunately, our patient accepted lumbar puncture at the beginning after admission and performed next-generation sequencing, which led us to a quick response for his treatment using the TMP-SMX. As a result, this patient had a significantly improved prognosis due to the rapid response treatment.

## 4. Conclusion

Our patient’s choroid plexitis, diffused meningitis, and suspected immune incompetence could be linked to the consideration of CNS nocardiosis. Next-generation sequencing, as well as CSF culturing, are the key to the diagnosis and can help physicians perform a rapid treatment and improve the prognosis. TMP-SMX is one of the optimal treatments for CSF Nocardiosis. Overall, patients with choroid plexitis combined with diffused meningitis and choroid plexitis are less reported in isolated CNS nocardiosis, which makes this case a significant reference for clinical practice.

## Acknowledgments

The authors thank the patient and his relatives for agreeing to report his case and providing a detailed medical history.

## Author contributions

**Conceptualization:** Qiujian Yu.

**Data curation:** Qiujian Yu, Ailan Pang, Shujuan Dai.

**Funding acquisition:** Qiujian Yu.

**Supervision:** Qiujian Yu.

**Writing**—**original draft:** Qiujian Yu.

**Writing**—**review & editing:** Qiujian Yu.

## References

[1] Ramachandran PS Wilson MR. Diagnostic testing of neurologic infections. Neurol Clin. 2018;36:687–703. 30366549 10.1016/j.ncl.2018.07.004 PMC6789377

[2] Duggal SD Chugh TD. Nocardiosis: a neglected disease. Med Princ Pract. 2020;29:514–23. 32422637 10.1159/000508717 PMC7768126

[3] Lucas SB Hounnou A Peacock C Beaumel A Kadio A De Cock KM. Nocardiosis in HIV-positive patients: an autopsy study in West Africa. Tuber Lung Dis. 1994;75:301–7. 7949078 10.1016/0962-8479(94)90137-6

[4] Bury K Citriniti V Bahrampour S Bajaj S Ferguson JF. Understanding the risk factors and pathogenesis of disseminated nocardiosis in immunocompromised patients. Cureus. 2024;16:e59838. 38846199 10.7759/cureus.59838 PMC11156491

[5] Nieves Perez CA Sánchez Pérez MJ Vargas AS Franco MA Molina Obana MC. Cerebral abscess due to *Nocardia beijingensis* associated with HIV: case report and mini review. Cureus. 2023;15:e47571. 38021684 10.7759/cureus.47571 PMC10666563

[6] Marak R Abdullah Behera M. Nocardiosis in kidney transplant recipients: a tertiary care center experience. Transpl Immunol. 2024;84:102041. 38537681 10.1016/j.trim.2024.102041

[7] Tian X Shi Q Liu P. Overlapping infection of *Nocardia farcinica* and *Aspergillus fumigatus* in a child with X-linked chronic granulomatous disease: a case report. BMC Infect Dis. 2022;22:69. 35057749 10.1186/s12879-021-06968-x PMC8772058

[8] Silwal S Mir M Boike S. Disseminated *Nocardia* brain abscess presenting as primary lung cancer with brain metastasis. Cureus. 2023;15:e43631. 37719483 10.7759/cureus.43631 PMC10504867

[9] Kanakan A Kumar A Kaur U. Case report: *Nocardia amamiensis* infection leading to worsening of chronic obstructive pulmonary disease symptoms in an elderly man. Am J Trop Med Hyg. 2023;109:1137–40. 37696514 10.4269/ajtmh.23-0284 PMC10622473

[10] Boctor N Aronowitz P. *Nocardia* brain abscess in a patient with diabetes: a case report. J Med Case Rep. 2023;17:336. 37553662 10.1186/s13256-023-04071-0 PMC10410777

[11] Ekrami A Khosravi AD Samarbaf Zadeh AR Hashemzadeh M. *Nocardia* co-infection in patients with pulmonary tuberculosis. Jundishapur J Microbiol. 2014;7:e12495. 25741428 10.5812/jjm.12495 PMC4335542

[12] Wilson JW. Nocardiosis: updates and clinical overview. Mayo Clin Proc. 2012;87:403–7. 22469352 10.1016/j.mayocp.2011.11.016 PMC3498414

[13] Wilson MR Naccache SN Samayoa E. Actionable diagnosis of neuroleptospirosis by next-generation sequencing. N Engl J Med. 2014;370:2408–17. 24896819 10.1056/NEJMoa1401268 PMC4134948

[14] Weng SS Zhang HY Ai JW. Rapid detection of *Nocardia* by next-generation sequencing. Front Cell Infect Microbiol. 2020;10:13. 32133300 10.3389/fcimb.2020.00013 PMC7040243

[15] Nasri E Fakhim H Barac A. *Nocardia farcinica* meningitis in a patient with high-grade astrocytoma. J Infect Dev Ctries. 2019;13:854–7. 32074098 10.3855/jidc.11582

[16] Kovoor JM Mahadevan A Narayan JP. Cryptococcal choroid plexitis as a mass lesion: MR imaging and histopathologic correlation. AJNR Am J Neuroradiol. 2002;23:273–6. 11847053 PMC7975266

[17] Jahid M Khan KU Rehan Ul H Ahmed RS. Overview of rheumatoid arthritis and scientific understanding of the disease. Mediterr J Rheumatol. 2023;34:284–91. 37941854 10.31138/mjr.20230801.oo PMC10628871

[18] Takahashi M Yamamoto J Idei M. Multiple intracranial nodules associated with rheumatoid arthritis: case report. Neurol Med Chir (Tokyo). 2014;54:317–20. 24140764 10.2176/nmc.cr2012-0259 PMC4533475

[19] Deigendesch N Costa Nunez J Stenzel W. Parasitic and fungal infections. Handb Clin Neurol. 2017;145:245–62. 28987173 10.1016/B978-0-12-802395-2.00018-3

[20] Beaman BL Beaman L. *Nocardia asteroides* as an invasive, intracellular pathogen of the brain and lungs. Subcell Biochem. 2000;33:167–97. 10804856 10.1007/978-1-4757-4580-1_8

[21] Hoshino Y Watanabe K Iida S. *Nocardia terpenica* sp. nov., isolated from Japanese patients with nocardiosis. Int J Syst Evol Microbiol. 2007;57(Pt 7):1456–60. 17625175 10.1099/ijs.0.64695-0

[22] Sher M Heard T Munckhof W Schwindack C. First case report of cerebral abscess from *Nocardia terpenica* and literature review. Clin Neurol Neurosurg. 2022;220:107341. 35779502 10.1016/j.clineuro.2022.107341

[23] Meena DS Kumar D Bohra GK Midha N Garg MK. Clinical characteristics and treatment outcome of central nervous system nocardiosis: a systematic review of reported cases. Med Princ Pract. 2022;31:333–41. 35700710 10.1159/000525509 PMC9485982

[24] Yuan D Shen L Qin BE. Central nervous system nocardiosis diagnosed by metagenomic next-generation sequencing: a case series and literature review. Adv Clin Exp Med. 2023;32:1453–63. 38112280 10.17219/acem/175818

